# Three Dose Levels of a Maternal Respiratory Syncytial Virus Vaccine Candidate Are Well Tolerated and Immunogenic in a Randomized Trial in Nonpregnant Women

**DOI:** 10.1093/infdis/jiab317

**Published:** 2021-06-19

**Authors:** Tino F Schwarz, Casey Johnson, Christine Grigat, Dan Apter, Peter Csonka, Niklas Lindblad, Thi Lien-Anh Nguyen, Feng F Gao, Hui Qian, Antonella N Tullio, Ilse Dieussaert, Marta Picciolato, Ouzama Henry

**Affiliations:** Institute of Laboratory Medicine and Vaccination Centre, Klinikum Würzburg Mitte, Campus Juliusspital, Würzburg, Germany; Johnson County Clin-Trials, Lenexa, Kansas, USA; Clinical Research Hamburg, Hamburg, Germany; VL-Medi, Helsinki, Finland; Centre for Child Health Research, Tampere University, Tampere, Finland; Terveystalo Turku Vaccine Clinic, Turku, Finland; GSK, Wavre, Belgium; GSK, Rockville, Maryland, USA; GSK, Rockville, Maryland, USA; GSK, Rockville, Maryland, USA; GSK, Rockville, Maryland, USA; GSK, Rixensart, Belgium; GSK, Rockville, Maryland, USA

**Keywords:** respiratory syncytial virus, maternal vaccine, immunogenicity, safety, nonpregnant women, vaccination, RSV, RSV vaccine

## Abstract

**Background:**

Respiratory syncytial virus (RSV) causes respiratory tract infections, which may require hospitalization especially in early infancy. Transplacental transfer of RSV antibodies could confer protection to infants in their first months of life.

**Methods:**

In this first-in-human, placebo-controlled study, 502 healthy nonpregnant women were randomized 1:1:1:1 to receive a single dose of unadjuvanted vaccine containing 30/60/120 µg of RSV fusion (F) protein stabilized in the prefusion conformation (RSVPreF3) or placebo.

**Results:**

Solicited local adverse events (AEs) were more frequently reported in the RSVPreF3 groups (4%–53.2%) versus placebo (0%–15.9%); most were mild/moderate. Unsolicited AEs were comparably reported among groups. Three serious AEs were reported; none was vaccination-related. Compared with prevaccination values, anti-RSV A neutralizing antibody geometric mean titers and anti-RSVPreF3 immunoglobulin G geometric mean concentrations increased 8- to 14-fold and 12- to 21-fold at day 8 and persisted 5- to 6-fold and 6- to 8-fold higher until day 91 in the RSVPreF3 groups versus 1-fold in placebo. Comparisons at day 8 and day 31 showed that the higher dose levels were significantly more immunogenic than the lowest one.

**Conclusions:**

The RSVPreF3 vaccine was well tolerated and immunogenic. The 60 and 120 µg dose levels were selected for further investigation in pregnant women.

**Clinical Trials Registration:**

NCT03674177.

## Teaser

In this first-in-human, placebo-controlled study, 30, 60, or 120 µg of unadjuvanted RSVPreF3 vaccine were well tolerated and immunogenic. Dose levels 60 and 120 µg were significantly more immunogenic than 30 µg and selected for further investigation in pregnant women.

Respiratory syncytial virus (RSV) causes lower respiratory tract infections (LRTI) leading to an estimated 3.2 million hospital admissions worldwide and 118 200 overall deaths in children younger than 5 years, especially in developing countries [1]. The incidence of severe disease requiring hospitalization is highest in infants under 6 months, and it is 3 times greater among premature compared to full-term infants under 1 year of age [1–3].

Despite the significant global health and financial burden of RSV infection, there are no licensed vaccines. Palivizumab and ribavirin are the solely available prophylactic and therapeutic options. However, the extensive use of palivizumab is hindered by the associated high costs, while the clinical benefit of ribavirin has not been conclusively established, and both therapies are only reserved for vulnerable groups at higher risk of severe RSV disease [4–7].

Current evidence suggests that vaccination of pregnant women against influenza, pertussis, diphtheria, and tetanus is safe, has an efficacy of up to 93% against infections in early infancy and provides protection to mothers and their infants [8, 9]. RSV vaccination during pregnancy is expected to boost serum neutralizing antibody responses induced by previous natural infections [10] and might reduce the incidence of RSV-associated LRTI in young infants by conferring passive immunity through placental antibody transfer [8, 9]. Previous studies investigating an RSV fusion (F) protein vaccine administered to pregnant women demonstrated that vaccine-induced RSV-specific antibodies were efficiently transferred from mother to infant [11–13].

The RSV F protein, which is highly conserved across RSV-A and RSV-B antigenic subgroups, is the main target of anti-RSV neutralizing antibody responses in human sera [14]. In addition, the prefusion conformation of the F protein exposes additional epitopes that elicit more potent neutralizing antibodies than the postfusion conformation [15, 16]. This led to the development of an RSV vaccine using the RSVPreF3 antigen, an engineered version of the F protein with a previously used sequence [17] stabilized in its prefusion conformation by introduction of cysteine residues, leading to the formation of a disulfide bond, and by filling of hydrophobic cavities [18, 19]. Immunogenicity and safety of RSVPreF3 were assessed in a series of preclinical studies, including 2 repeat-dose studies (rabbits) and 2 developmental and reproductive toxicity studies (1 in rabbits and 1 in rats), which indicated that the RSVPreF3 vaccine candidate was well tolerated at the proposed doses.

**Figure 1.**
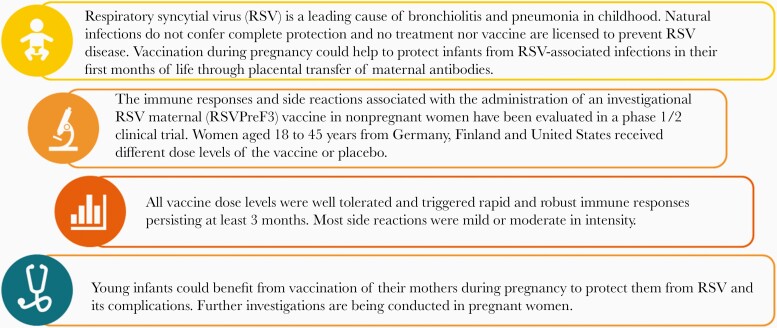
Plain language summary.

This first-in-human study evaluated the safety, reactogenicity, and immunogenicity of the maternal RSVPreF3 vaccine candidate administered to nonpregnant women of childbearing age, at 3 different dose levels, as compared with placebo. A plain language summary contextualizing the relevance, the results, and the impact of our study is described in Figure 1.

## METHODS

### Study Design and Participants

This phase I/II, randomized, placebo-controlled, observer-blind, first-in-human trial was conducted between October 2018 and September 2019 at 2 centers in the United States, 4 in Finland, and 5 in Germany. Healthy nonpregnant women 18–45 years of age were enrolled in the study after providing informed consent. Women of childbearing potential were willing to practice adequate contraception for 30 days prior to vaccination, had a negative pregnancy test on the day of vaccination, and agreed to continue adequate contraception up to 90 days postvaccination. Women who previously received an investigational RSV vaccine or any vaccine within 30 days before and after study vaccination (except inactivated influenza vaccine, which could be administered up to 15 days before or from 15 days after study vaccination) were excluded.

Women were randomized in a 1:1:1:1 ratio to receive a single dose of either 30 µg (30 RSVPreF3 group), 60 µg (60 RSVPreF3 group), or 120 µg (120 RSVPreF3 group) of unadjuvanted RSVPreF3 vaccine, or placebo (placebo group). Women were vaccinated on day 1 and had 4 additional clinic visits (days 8, 31, 61, and 91) and 1 follow-up phone call (day 181 [study end]).

All 3 dose levels of the study vaccine were presented in single-dose vials as lyophilized RSVPreF3 antigen and were reconstituted in 150 mM sodium chloride solution to obtain 30 µg, 60 µg, and 120 µg of antigen per 0.5 mL dose. Placebo was administered as a 0.5 mL dose of 150 mM sodium chloride solution. Study vaccine and placebo were administered intramuscularly in the deltoid region of the nondominant arm.

As this was the first administration of the unadjuvanted RSVPreF3 vaccine in humans, enrollment and vaccination were conducted in 2 sequential steps monitored by a blinded safety review team and by an unblinded internal safety review committee, not otherwise involved in the current study. During the first step, 60 women were enrolled and randomized across the 4 study groups, only at United States sites. Vaccination was limited to a total of 10 women per day across sites until the first 30 women had been sequentially vaccinated at least 60 minutes apart. Enrollment and vaccination in the second step were contingent on the lack of an observed safety signal during each of the 2 safety reviews performed by the safety review team and the internal safety review committee on days 8 and 31.

Women were randomized using a centralized randomization system on the Internet. The randomization algorithm used a minimization procedure accounting for age and center. The study was observer blind up to day 91, after which the statistician and data management staff had access to the individual woman treatment assignments and the study was conducted in a single-blinded manner. Study participants, site investigators, laboratories, and study personnel responsible for assessing the study endpoints remained blinded to treatment allocation up to study end.

The study was registered on ClinicalTrials.gov (NCT03674177) and was conducted in accordance with the principles of Good Clinical Practice, the Declaration of Helsinki, and all applicable regulatory requirements. The full protocol of this study is available at https://www.gsk-studyregister.com (ID 208068). Anonymized individual participant data and study documents can be requested for further research at https://www.clinicalstudydatarequest.com.

### Objectives

The primary objective was to evaluate the safety and reactogenicity of the 30, 60, and 120 µg dose levels of the RSVPreF3 investigational vaccine versus placebo up to 1 month postvaccination (day 31). Secondary objectives were to evaluate the safety of the 30, 60, and 120 µg dose levels of the RSVPreF3 investigational vaccine versus placebo up to 6 months postvaccination (day 181) and the humoral response to RSV antigenic subgroup A of the 30, 60, and 120 µg dose levels of the RSVPreF3 investigational vaccine versus placebo up to 3 months postvaccination (day 91). Tertiary objectives (including humoral response to RSV antigenic subgroup B) are not presented here.

### Reactogenicity and Safety Assessment

All women were observed for 60 minutes postvaccination for any reactions. Solicited local and general adverse events (AEs) were recorded on diary cards for a 7-day and unsolicited AEs for a 30-day postvaccination period. Symptom intensity was graded as 0 (none, used only for solicited AEs), 1 (mild), 2 (moderate), and 3 (severe). Grade 3 was defined as significant injection site pain at rest, redness and/or swelling at the injection site of >100 mm diameter, fever of >39°C, and preventing normal everyday activities for all the other AEs.

**Figure 2.**
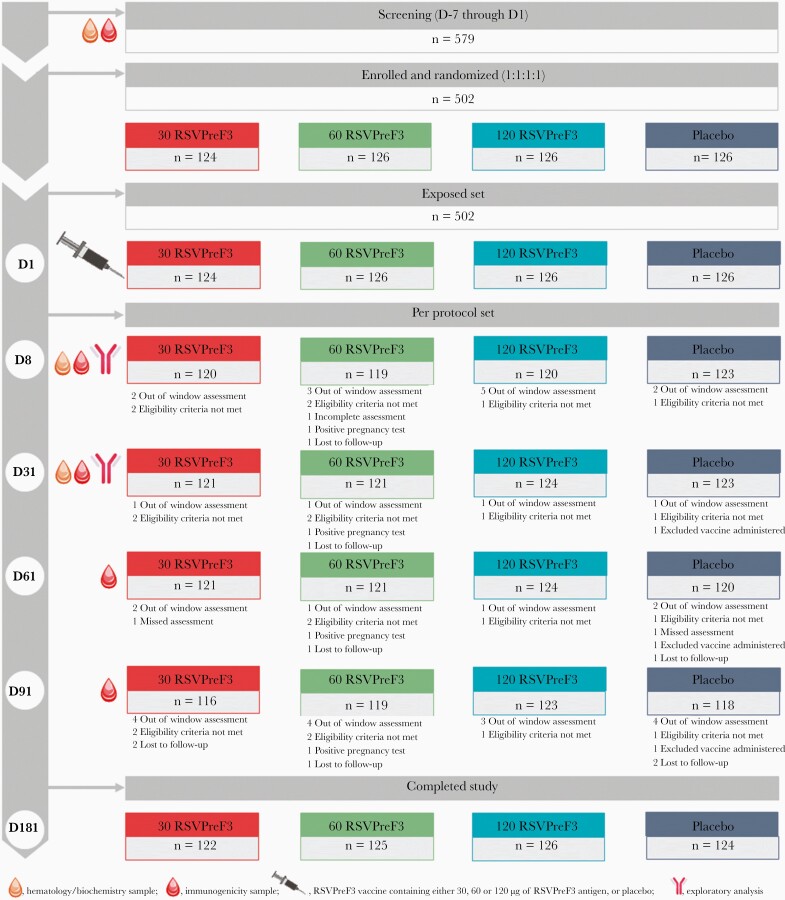
Participant flow chart. Abbreviations: 30 RSVPreF3/60 RSVPreF3/120 RSVPreF3, group receiving 1 dose of the RSV vaccine containing 30, 60, or 120 µg of RSVPreF3 antigen; D, day; n, number of women; placebo, group receiving 1 dose of placebo; RSV, respiratory syncytial virus.

Medically attended solicited and unsolicited AEs were collected. Serious AEs (SAEs), AEs leading to withdrawal, and occurrence of pregnancies were collected from day 1 to day 181. Hematological and biochemical parameters were measured at the screening visit, and days 8 and 31 postvaccination (Figure 2).

### Immunogenicity Assessment

Approximately 30 mL of blood were collected from participating women at the screening visit, and at days 8, 31, 61, and 91 postvaccination to assess anti-RSV A neutralizing antibodies geometric mean titers (GMTs) and anti-RSVPreF3 immunoglobulin G (IgG) geometric mean concentrations (GMCs) (Figure 2).

Anti-RSV A neutralizing antibodies were measured using an in-house RSV serum neutralization assay at GSK laboratory, Wavre, Belgium. The RSV-infected cells were detected using a primary antibody directed against RSV (anti-RSV IgG) and a secondary antibody conjugated with horseradish peroxidase, allowing the visualization of plaques after coloring. Serum neutralizing antibodies titers, expressed as the estimated dilution 60 (ED60), corresponded to the inverse of the interpolated serum dilution inducing a 60% reduction in the number of plaques compared to the virus placebo wells. The assay cutoff was 18 ED60.

Anti-RSVPreF3 IgG antibodies were measured using an enzyme-linked immunosorbent assay (ELISA) at Nexelis Labs, Laval, Quebec, Canada. The assay cutoff was 25 ELISA units (EU)/mL. Additional details are provided in the Supplementary Material.

### Statistical Analysis

A planned sample size of 125 women per group was justified based on precision estimation on safety and immunogenicity:

1. Safety considerations: with 125 women per group, if an AE is not observed in a given group, the true incidence rate of the AE in that group is ≤2.9% with 95% confidence.
2. Immunogenicity considerations: with 125 women per group, if the geometric mean ratio of 1.5 for RSV-A neutralizing antibodies between 2 dose groups is observed, the 95% confidence interval (CI) would be 1.19–1.89 based on a standard deviation of 0.4 of log10 transformed RSV-A neutralizing antibodies.

All statistical analyses were performed using SAS Drug Development software.

The reactogenicity and safety analyses were performed on the exposed set and included all women with documented vaccination. Percentages of women reporting solicited and unsolicited AEs were tabulated with exact 95% CIs. Same computations were performed for AEs of ≥ grade 2, AEs considered as vaccination related (all and grade 3), and medically attended AEs (all and grade 3). SAEs and occurrence of pregnancies were described. Toxicity of hematological and biochemical parameters was graded from 1 to 4 using the Food and Drug Administration Guidance [20].

The immunogenicity analysis was performed on the per-protocol set and included all women who met eligibility criteria, received the study vaccine or placebo, complied with protocol-defined procedures, and had immunogenicity results for at least 1 assay at the corresponding time point. Exploratory comparisons between groups were performed for anti-RSV A neutralizing antibody GMTs and anti-RSVPreF3 IgG antibody GMCs at day 8 (post hoc analysis) and at day 31 (prespecified) using an analysis of covariance (ANCOVA) model with vaccine group as fixed effect and prevaccination titer/concentration as covariate. Pairwise comparisons were made using the Tukey multiple comparison adjustment. Anti-RSV A neutralizing antibodies GMTs, anti-RSVPreF3 IgG antibody GMCs, and seropositivity rates were calculated with 95% CIs. GMTs and GMCs were determined by taking the anti-log of the mean of the log titer transformations. For results below the assay cutoff, an arbitrary value of half the cutoff was considered for calculation of GMTs, GMCs, and fold increase.

## RESULTS

### Participants

A total of 502 women were enrolled and vaccinated (124 in the 30 RSVPreF3 group, 126 in the 60 RSVPreF3 group, 126 in the 120 RSVPreF3 group, and 126 in the placebo group). Of these, 497 women completed the study, and 5 women were lost to follow-up. There were no withdrawals due to an AE (Figure 2). Groups were well balanced in terms of baseline characteristics (Table 1).

**Table 1.** Baseline Characteristics of Study Participants, Exposed Set

Characteristic	30 RSVPreF3 (n = 124)	60 RSVPreF3 (n = 126)	120 RSVPreF3 (n = 126)	Placebo (n = 126)
Age in years at first vaccination, mean (SD)	32.5 (7.4)	32.1 (7.9)	31.5 (7.6)	32.2 (7.1)
Age group, n (%)				
18–32, y	62 (50)	64 (50.8)	64 (50.8)	66 (52.4)
33–45, y	62 (50)	62 (49.2)	62 (49.2)	60 (47.6)
Country, n (%)				
Finland	15 (12.1)	15 (11.9)	16 (12.7)	15 (11.9)
Germany	52 (41.9)	54 (42.9)	52 (41.3)	53 (42.1)
United States	57 (46.0)	57 (45.2)	58 (46.0)	58 (46.0)
Ethnicity, n (%)				
Hispanic or Latino	5 (4.0)	2 (1.6)	2 (1.6)	2 (1.6)
Not Hispanic or Latino	119 (96.0)	124 (98.4)	124 (98.4)	124 (98.4)
Race, n (%)				
Asian	1 (0.8)	1 (0.8)	2 (1.6)	5 (4.0)
Black or African American	5 (4.0)	5 (4.0)	4 (3.2)	7 (5.6)
White	115 (92.7)	120 (95.2)	117 (92.9)	114 (90.5)
Other	3 (2.4)	0 (0)	3 (2.4)	0 (0)

Abbreviations: 30 RSVPreF3/60 RSVPreF3/120 RSVPreF3, group receiving 1 dose of RSV vaccine containing 30, 60, or 120 µg of RSVPreF3 antigen; n, number of women; placebo, group receiving 1 dose of placebo; RSV, respiratory syncytial virus.

### Safety and Reactogenicity

**Figure 3.**
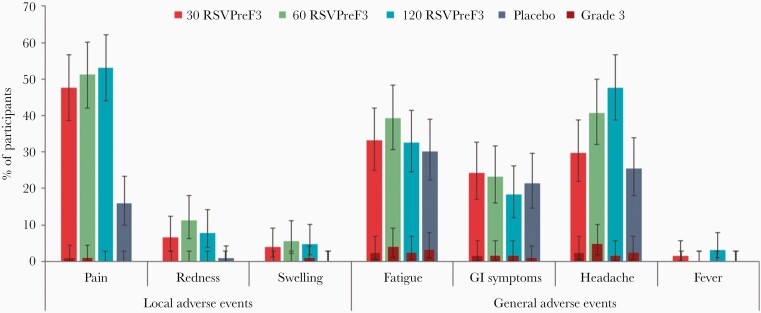
Incidence of solicited local and general adverse events from day 1 to day 7 postvaccination (exposed set). Error bars indicate 95% confidence intervals. Abbreviations: 30 RSVPreF3/60 RSVPreF3/120 RSVPreF3, group receiving 1 dose of the RSV vaccine containing 30, 60, or 120 µg of RSVPreF3 antigen; GI, gastrointestinal; placebo, group receiving 1 dose of placebo; RSV, respiratory syncytial virus.

Pain at the injection site was the most common solicited local AE, reported by 47.6%–53.2% of women in the RSVPreF3 groups versus 15.9% in the placebo group. Redness was reported by ≤11.2% of women in the RSVPreF3 groups versus 0.8% in the placebo group. Swelling was reported only in the RSVPreF3 groups (≤5.6%). Headache was the most common solicited general AE, reported by 29.8%–47.6% of women in the RSVPreF3 groups versus 25.4% in the placebo group, followed by fatigue and gastrointestinal symptoms (≤39.2% and ≤24.2% across all groups). Fever was reported infrequently and only in the RSVPreF3 groups (≤3.2% of women [2 in 30 RSVPreF3 group and 4 in 120 RSVPreF3 group]). Of these, 1 woman reported a temperature >38.5–39°C (120 RSVPreF3 group). No grade 3 fever was reported. Overall, grade 3 solicited AEs were reported by ≤4.8% of women in all groups (Figure 3). The average duration of solicited local and general AEs was approximately 1–3 days in all groups, with a median duration of 2 days for injection site pain and 1–2 days for headache.

Unsolicited AEs reports were comparable among all groups (36.3%–38.1% of women in the RSVPreF3 groups vs 34.9% in the placebo group). Headache, after the 7-day postvaccination period, was the most frequently reported unsolicited AE (≤8.1% in RSVPreF3 groups vs ≤11.1% in the placebo group). The incidence of unsolicited AEs considered related to vaccination was 6.5%–11.1% in the RSVPreF3 groups versus 7.9% in the placebo group. Grade 3 unsolicited AEs were infrequently reported (2.4%–7.9% in the RSVPreF3 groups vs 1.6% in the placebo group). One grade 3 unsolicited AE (myalgia, ie, muscular pain) reported in the 60 RSVPreF3 group was considered as vaccination related and was resolved within 2 days. The majority of unsolicited AEs occurring within 30 days postvaccination lasted 1–7 days, with a 1-day median duration for headache.

There were 3 medically attended solicited AEs (fatigue, gastrointestinal symptoms, and headache) experienced by the same woman in the 30 RSVPreF3 group. The incidence of medically attended unsolicited AEs was 4.0%–7.9% in RSVPreF3 groups versus 6.3% in the placebo group. Three SAEs were reported throughout the duration of the study (1 in the 120 RSVPreF3 group and 2 in the placebo group). All occurred after more than 60 days following vaccination and were resolved within 20 days; none was considered as vaccine related by the investigator. Two pregnancies occurred during the study, 1 in the 60 RSVPreF3 group that started around the time of vaccination and 1 in the 120 RSVPreF3 group that started approximately 4 months following vaccination. For both pregnancies, the outcome was a live infant with no apparent congenital anomalies.

No occurrences of clinically significant changes in laboratory parameters were observed. At day 8, one woman (30 RSVPreF3 group) had grade 3 increase in creatinine (laboratory error), and another woman (60 RSVPreF3 group) had grade 3 increase in alanine and aspartate aminotransferase counts; both women had normal values at baseline. Two women (60 RSVPreF3 group) had grade 3 decrease in hemoglobin count from baseline, 1 at day 8 (considered related to vaccination by the investigator and resolved by day 31) and the other 1 at day 31. Both values remained within normal ranges and grade 0 based on the Food and Drug Administration toxicity scale. One woman (30 RSVPreF3 group), for whom a normal value was observed at baseline, had grade 3 change in neutrophil count at day 31 (considered unrelated to the vaccination and possibly attributable to a concomitant cold).

### Immunogenicity

**Figure 4.**
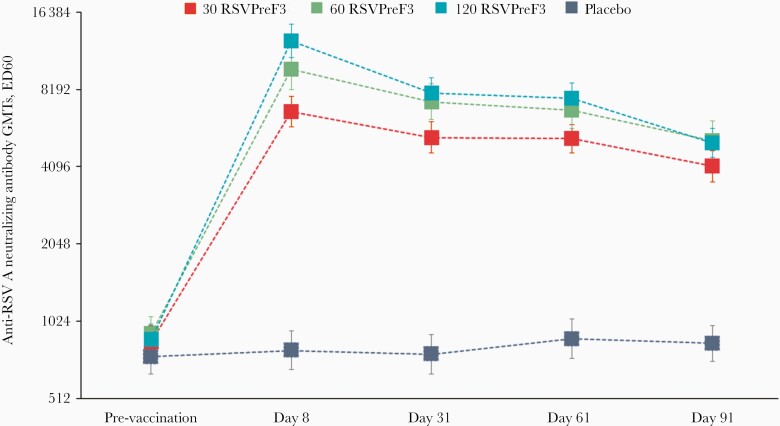
Anti-RSV A neutralizing antibody geometric mean titers (neutralization assay [ED60]; per protocol set). Error bars indicate 95% confidence intervals. Abbreviations: 30 RSVPreF3/60 RSVPreF3/120 RSVPreF3, group receiving 1 dose of the RSV vaccine containing 30, 60, or 120 µg of RSVPreF3 antigen; ED60, serum dilution inducing 60% reduction in plaque-forming units; GMTs, geometric mean titers; placebo, group receiving 1 dose of placebo; RSV, respiratory syncytial virus.

All women had anti-RSV A neutralizing antibodies titers equal to or above the seropositivity cutoff at baseline (Figure 4). Compared to prevaccination titers, anti-RSV A neutralizing antibodies GMTs were boosted in all RSVPreF3 groups with a rapid response peaking at day 8 postvaccination. Fold increases of 7.82 in the 30 RSVPreF3 group, 10.79 in the 60 RSVPreF3 group, 14.28 in the 120 RSVPreF3 group versus 1.06 in the placebo group were noted. At day 91 postvaccination, anti-RSV A neutralizing antibodies GMTs had declined in all RSVPreF3 groups (Figure 4) but remained 4.84-fold (30 RSvPreF3 group) to 5.88-fold (120 RSVPreF3 group) higher than prevaccination values.

Between-group exploratory comparisons of anti-RSV A neutralizing antibodies GMTs at day 8 and day 31 showed that the 60 and 120 RSVPreF3 groups were statistically more immunogenic than the 30 RSVPreF3 group. Although not statistically significant, a trend towards higher immune response for the 120 RSVPreF3 group compared to that for the 60 RSVPreF3 group was observed (Supplementary Table 1).

**Figure 5.**
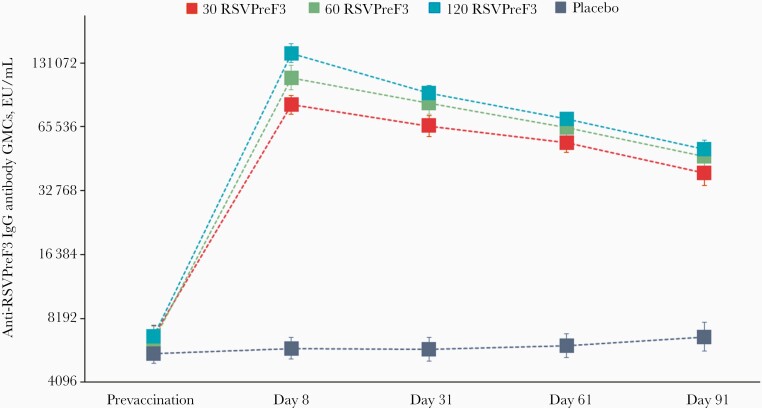
Anti-RSVPreF3 IgG antibody geometric mean concentrations (enzyme-linked immunosorbent assay [EU/mL]; per protocol set). Error bars indicate 95% confidence intervals. Abbreviations: 30 RSVPreF3/60 RSVPreF3/120 RSVPreF3, group receiving 1 dose of the RSV vaccine containing 30, 60, or 120 µg of RSVPreF3 antigen; EU, laboratory units of enzyme-linked immunosorbent assay; GMCs, geometric mean concentrations; IgG, immunoglobulin G; placebo, group receiving 1 dose of placebo; RSV, respiratory syncytial virus.

Anti-RSVPreF3 IgG antibodies GMCs also peaked at day 8 in all RSVPreF3 groups, with fold increases of 12.16 in the 30 RSVPreF3 group, 17.58 in the 60 RSVPreF3 group, 21.25 in the 120 RSVPreF3 group versus 1.05 in the placebo group. The immune responses declined slowly over the following time points and persisted 5.96-fold (30 RSVPreF3) to 7.61-fold (120 RSVPreF3) above prevaccination values at day 91 (Figure 5).

As for the anti-RSV A neutralizing antibodies, between-group ANCOVA analyses of anti-RSVPreF3 IgG antibodies GMCs at day 8 and day 31 showed that the higher RSVPreF3 dose levels were statistically more immunogenic than the 30 RSVPreF3 one. Furthermore, the exploratory comparison at day 8, but not at day 31, showed that the 120 RSVPreF3 group was statistically more immunogenic than the 60 RSVPreF3 group (Supplementary Table 1).

## DISCUSSION

This first-in-human study of a maternal RSVPreF3 vaccine candidate, containing either 30, 60, or 120 µg of RSVPreF3 antigen, showed that all dose levels were well tolerated and boosted efficiently preexisting humoral immune responses to RSV in nonpregnant women of childbearing age. The incidence of AEs was comparable between vaccine groups, with most AEs being mild or moderate in intensity. Among solicited AEs, although headache incidence tended to increase with increasing dose level, grade 3 headache was similarly reported among RSVPreF3 and placebo groups. Fever was mostly uncommon and of low grade. These reassuring reactogenicity results are encouraging for a vaccine that is intended to be used in pregnant women, as maternal fever during pregnancy has been associated with adverse child outcomes, such as neural tube defects, congenital heart defects, oral clefts, or renal anomalies [21–24].

As expected, all women were seropositive for anti-RSV A neutralizing antibodies and anti-RSVPreF3 IgG antibodies due to lifelong priming with RSV. Vaccination with RSVPreF3 induced a rapid and robust humoral immune response for all the RSVPreF3 dose levels, peaking at day 8. GMTs of anti-RSV A neutralizing antibodies and GMCs of anti-RSVPreF3 IgG antibodies declined between day 8 and day 91 with comparable kinetics in all RSVPreF3 groups but persisted well above prevaccination concentrations at day 91. Vaccination during the second or third trimester of pregnancy has been shown to be an effective strategy to protect infants during the period of great susceptibility to infectious diseases, when they are too young to be protected through routine pediatric vaccination and unable to mount an optimum immune response against certain pathogens [9, 10, 25]. High titers of maternally derived RSV neutralizing antibodies have been shown to be associated with subsequent serologic protection of infants against RSV infection and lower incidence of RSV-associated acute LRTI during the first 6 months of life [26–30]. Administration of an RSV vaccine to pregnant women is expected to boost the serum neutralizing antibody response induced by previous natural infections and has the potential to prevent or reduce the severity of RSV disease and RSV-associated LRTI hospitalizations in infants in their first weeks or months of life [11–13, 31, 32].

The optimal time window to vaccinate during pregnancy should balance out early enough vaccination (to achieve the peak immune response), timing of the most efficient transfer of antibodies to the infant, expected time of delivery, and postpartum antibody persistence. Trials with influenza and pertussis vaccines suggested that the optimal transfer of maternal antibodies may occur upon vaccination towards the beginning of the third trimester of pregnancy for infants born at full-term but may not be optimal for preterm infants [33–37]. The rapid increase in RSV A neutralizing antibody titers observed with RSVPreF3 may provide flexibility for mothers to get vaccinated while still allowing for a substantial transfer of antibodies to their infants. Moreover, boosted antibodies induced by RSVPreF3 remained well above baseline levels at 3 months postvaccination, suggesting that RSVPreF3 might be administered earlier during pregnancy without impacting the level of antibodies transferred to the infants.

Exploratory analysis at days 8 and 31 showed that the 60 RSVPreF3 and 120 RSVPreF3 dose levels were more immunogenic than the 30 RSVPreF3 one. Additionally, a trend towards higher immune response was noted for the 120 RSVPreF3 dose level, but this did not reach statistical significance for all readouts, with the exception of day 8 ANCOVA results for anti-RSVPreF3 IgG antibodies. These results are supporting further evaluation of the vaccine’s higher doses.

Previous studies evaluating the impact of adding aluminum into the formulation of an investigational RSV F protein vaccine engineered to preferentially maintain the prefusion conformation have shown that the presence of aluminum adjuvant did not enhance the immune response, while significantly contributing to higher rates of grade 2/3 AEs and/or fever [38, 39]. Our study results show that each of the 3 dose levels were able to induce robust neutralizing antibodies, despite the fact that the vaccine did not contain an aluminum adjuvant.

A limitation of the study is that the vast majority of the participants were white, Caucasian women, which may limit the generalizability of the results to the broader population. Another limitation is the presence of a potential boosting of the humoral response due to natural exposure to RSV infection during study period enrolment or collection of blood samples, which has been mostly surpassed by the controlled study design. Additionally, all comparative analyses were descriptive and should be interpreted with caution, as no adjustment for multiplicity was performed.

## CONCLUSIONS

The RSVPreF3 vaccine was well tolerated and no safety concerns were identified. All dose levels were immunogenic, with higher immune response induced by the 60 and 120 µg dose levels than the 30 µg one. These data support further investigation of the 60 and 120 µg RSVPreF3 dose levels in pregnant women.

## Supplementary Data

Supplementary materials are available at *The Journal of Infectious Diseases* online. Consisting of data provided by the authors to benefit the reader, the posted materials are not copyedited and are the sole responsibility of the authors, so questions or comments should be addressed to the corresponding author.

jiab317_suppl_Supplementary_Materials

## Notes

***Acknowledgments.*** We thank the women who took part in this study as well as the staff at the participating institutions, the members of the GSK safety review committee, and GSK local delivery and study teams. We also thank Modis c/o GSK: Andrada-Roxana Pascu provided medical writing services and Quentin Deraedt provided editorial assistance and coordinated the publication development.

***Author contributions.*** T. F. S. carried out investigations and original draft preparation. C. J., C. G., D. A., P. C., and N. L. carried out investigations, and manuscript review and editing. T. L.-A. N. and A. N. T. performed formal analysis, validation, and manuscript review and editing. F. F. G. and H. Q. contributed to methodology, formal analysis, validation, and manuscript review and editing. I. D. and O. H. contributed to conceptualization, methodology, project administration, supervision, and manuscript review and editing. M. P. contributed to conceptualization, methodology, project administration, supervision, and original draft manuscript preparation.

***Financial support.*** This work was supported by GlaxoSmithKline Biologicals SA. GlaxoSmithKline Biologicals SA was involved in all stages of the study conduct and analysis, and took responsibility for all costs associated with the development and publishing of the present manuscript.

***Potential conflicts of interest***. T. F. S. reports personal fees from the GlaxoSmithKline group of companies (GSK), Pfizer, Biogen, Merck Serono, Sanofi, Biotest, Sequirus, and Bavarian Nordic outside the submitted work. Institutions of D. A. and P. C. received grants/fees from GSK for conducting the present work. T. L.-A. N., F. F. G., H. Q., A. N. T., I. D., M. P., and O. H. are/were GSK employees at the time of the study conduct. T. L.-A. N., F. F. G., A. N. T., I. D., M. P., and O. H. receive GSK shares/stock options as part of their former/current employee remuneration. All other authors report no potential conflicts of interest. All authors have submitted the ICMJE Form for Disclosure of Potential Conflicts of Interest. Conflicts that the editors consider relevant to the content of the manuscript have been disclosed.
