# Supplementary material for: Three Dose Levels of a Maternal Respiratory Syncytial Virus Vaccine Candidate Are Well Tolerated and Immunogenic in a Randomized Trial in Nonpregnant Women
Source: J Infect Dis. 2021 Jun 19;225(12):2067–76. doi: 10.1093/infdis/jiab317 (PMC9200160; doi:10.1093/infdis/jiab317)
Supplement: jiab317_suppl_Supplementary_Materials [file jiab317_suppl_supplementary_materials.docx]

**Title:** **Three dose levels of a maternal respiratory syncytial virus vaccine candidate are well tolerated and immunogenic in a randomized trial in non-pregnant women**

**Supplemental methods**

**RSV A/B neutralization assay**

The serum neutralization assay is a functional assay that measures the ability of serum antibodies to neutralize RSV entry and replication in a host cell line. First, virus neutralization is performed by incubating a fixed amount of RSV-A strain (Long) or RSV-B strain (B18537) with serial dilutions of the test serum. Then, the serum/virus mixture is transferred onto a monolayer of Vero cells (African Green Monkey, kidney, Cercopitheus aethiops, ATCC CCL-81) and incubated for two days to allow infection and the formation of plaques by non-neutralized viruses. Following the fixation period, RSV-infected cells are detected using a primary antibody directed against RSV (anti-RSV IgG) and a secondary antibody conjugated with horse-radish peroxidase (HRP), allowing the visualization of plaques after coloration with TrueBlue^TM^ peroxidase substrate. Viral plaques are counted using an automated microscope coupled to an image analyzer (Scanlab system with Axiovision software). For each serum dilution, a ratio, expressed as a percentage, is calculated between the number of plaques at that dilution and the number of plaques in the virus control wells (no serum added). The serum Nab titers is expressed in ED60 (Estimated Dilution 60) and corresponds to the inverse of the interpolated serum dilution that yields a 60% reduction in the number of plaques compared to the virus control wells as described by others [1, 2].

**RSVPreF3 ELISA**

The RSVPreF3 IgG ELISA is based on an indirect ELISA allowing the detection and the quantification of total IgG antibodies directed against RSVPreF3 in human serum samples. The principle of this assay is as follows: RSVPreF3 antigen is adsorbed onto a 96-well polystyrene microplate. After a washing and a blocking step, dilutions of serum samples, controls and standards are added to the coated microplate. A reference standard curve is prepared using a pool of commercial human serum containing anti-RSV antibodies. After incubation, the microplate is washed to remove unbound primary antibodies. Bound IgG is detected by the addition of a secondary anti-human antibody conjugated to HRP. Bound antibodies are quantified by the addition of the HRP substrate, tetramethylbenzidine and hydrogen peroxide, whereby a colored product develops proportionally to the amount of anti-RSVPreF3 IgG antibodies present in the serum sample. The optical density of each sample dilution is then interpolated on the reference standard. The corresponding antibody concentration, corrected for the dilution factor, is expressed in arbitrary ELISA Laboratory Units per milliliter (EU/mL).”

**References**:

1. Barbas CF, Crowe JE, Cabada D, et al. Human monoclonal Fab fragments derived from a combinatorial library bind to respiratory syncytial virus F glycoprotein and neutralize infectivity. Proc Natl Acad Sci USA **1992**; 89:10164–68.
2. Bates JT, Keefer CJ, Slaughter JC, et al. Escape from neutralization by the respiratory syncytial virus-specific neutralizing monoclonal antibody palivizumab is driven by changes in on-rate of binding to the fusion protein. Virology **2014**; 454–455:139–44.

**Supplementary Table 1**. Exploratory comparisons between RSVPreF3 groups for anti-RSV A neutralizing antibody geometric mean titers and anti-RSVPreF3 IgG antibody geometric mean concentrations at days 8 and 31 (per protocol set)

|  | Anti-RSV A neutralizing antibody GMTs | | | |  | Anti-RSVPreF3 IgG antibody GMCs | | | |
| --- | --- | --- | --- | --- | --- | --- | --- | --- | --- |
|  | Day 8 | | Day 31 | |  | Day 8 | | Day 31 | |
|  | N | value (95% CI) | N | value (95% CI) |  | N | value (95% CI) | N | value (95% CI) |
| *Adjusted GMT or GMC* |  |  |  |  |  |  |  |  |  |
| 30 RSVPreF3 | 119 | 6447.50 | 120 | 5425.92 |  | 119 | 80506.29 | 120 | 62929.72 |
| 60 RSVPreF3 | 118 | 9177.77 | 120 | 7133.69 |  | 118 | 110093.97 | 120 | 82950.72 |
| 120 RSVPreF3 | 120 | 11916.66 | 124 | 7932.35 |  | 120 | 140910.73 | 124 | 89760.38 |
| *GMT or GMC ratio* |  |  |  |  |  |  |  |  |  |
| 120/30 RSVPreF3 |  | 1.85 (1.41–2.43) |  | 1.46 (1.15–1.86) |  |  | 1.75 (1.42–2.15) |  | 1.43 (1.17–1.74) |
| 120/60 RSVPreF3 |  | 1.30 (0.99–1.71) |  | 1.11 (0.87–1.42) |  |  | 1.28 (1.04–1.57) |  | 1.08 (0.89–1.32) |
| 60/30 RSVPreF3 |  | 1.42 (1.08–1.87) |  | 1.31 (1.03–1.68) |  |  | 1.37 (1.11–1.68) |  | 1.32 (1.08–1.61) |

Abbreviations: CI, confidence interval; GMC, geometric mean concentration; GMT, geometric mean titer; IgG, immunoglobulin G; N, number of women with both pre- and post-vaccination results available; 30 RSVPreF3/60 RSVPreF3/120 RSVPreF3, group receiving 1 dose of the respiratory syncytial virus (RSV) vaccine containing 30, 60 or 120 µg of RSVPreF3 antigen
